# Physical activity and sedentary behavior as multimorbidity discriminators among elderly Brazilians: a cross-sectional study

**DOI:** 10.1590/1516-3180.2020.0504.R1.1802021

**Published:** 2021-06-25

**Authors:** Marina Christofoletti, Paula Fabrício Sandreschi, Emanuele Naiara Quadros, Eleonora d’Orsi, Cassiano Ricardo Rech, Sofia Wolker Manta, Tânia Rosane Bertoldo Benedetti

**Affiliations:** I MSc. Doctoral Student, Department of Physical Education, Universidade Federal de Santa Catarina (UFSC), Florianópolis (SC), Brazil; II MSc. Doctoral Student, Department of Physical Education, Universidade Federal de Santa Catarina (UFSC), Florianópolis (SC), Brazil.; III Master’s Student, Department of Physical Education, Universidade Federal de Santa Catarina (UFSC), Florianópolis (SC), Brazil.; IV PhD. Full Professor, Department of Public Health, Universidade Federal de Santa Catarina (UFSC), Florianópolis (SC), Brazil.; V PhD. Full Professor, Department of Physical Education, Universidade Federal de Santa Catarina (UFSC), Florianópolis (SC), Brazil.; VI MSc. Doctoral Student, Department of Physical Education, Universidade Federal de Santa Catarina (UFSC), Florianópolis (SC), Brazil.; VII PhD. Full Professor, Department of Physical Education, Universidade Federal de Santa Catarina (UFSC), Florianópolis (SC), Brazil.

**Keywords:** Healthy lifestyle, Exercise, Sedentary behavior, Chronic disease, Epidemiology, Healthy behavior, Physical activity, Lifestyle, sedentary, Chronic illness

## Abstract

**BACKGROUND:**

Associations between behaviors and individual chronic diseases have been demonstrated. However, the relationship between time spent on sedentary behavior and multimorbidity remains less clear.

**OBJECTIVE:**

To identify the predictive power of various intensities of physical activity versus sedentary behavior, as discriminatory factors for cardiometabolic multimorbidity (cardiovascular diseases and diabetes) in the elderly.

**DESIGN AND SETTING:**

Cross-sectional study in different residential census tracts and residential households in Florianópolis (SC).

**METHODS:**

The participants were 425 elderly people (65% women) from the EpiFloripa Aging study in 2014. Sociodemographic variables and self-reported incidence of cardiovascular diseases and diabetes were obtained via a questionnaire. Light physical activity (LPA), moderate-to-vigorous physical activity (MVPA) and sedentary behavior (SB) were measured using accelerometers. The analyses were stratified according to sex and included a diagnosis for interpretation. Behaviors were taken into consideration if their predictive power in terms of area under the receiver operating characteristic (ROC) curve was greater than 0.50. The time cutoff point was defined from sensitivity and specificity.

**RESULTS:**

For older adult men with diabetes, the predictive value of MVPA for absence of multi-morbidity was an area of 0.75 (95% confidence interval, CI: 0.538-0.962), and a cutoff of 17 minutes per day. Older adult women with diabetes had an area of 0.71 (95% CI: 0.524-0.866) and a cutoff of 10 minutes per day. LPA and SB did not present predictive values.

**CONCLUSION:**

The time spent on MVPA is a predictor of absence of multimorbidity in elderly people with diabetes, for both sexes.

## INTRODUCTION

Multimorbidity is the existence of two or more diseases simultaneously. Its presence may lead to a 5.5-fold higher public healthcare cost per patient.^1^ Involvement of multimorbidity, such as cardiovascular disease and concomitant diabetes, has higher prevalence among the elderly, and is the main cause of hospitalizations in Brazil.^2^^,^^3^ Diabetes combined with hypertension increases the chances of having coronary heart disease and/or cerebrovascular disease threefold, compared with presence of diabetes alone.^4^ This reflects mortality rates for all causes, given that the prevalence of hypertension grows at population levels.^5^ Among the consequences of multimorbidities in the elderly are the risk of falls, depression and declines in physical function performance and in daily activities.^6^

Hence, reduction of the prevalence of these diseases is included in the agendas of international governmental bodies, such as the Global Strategy on Healthy Eating, Physical Activity and Health^7^ and, in Brazil, the Strategic Action Plan for the Fight against Chronic Noncommunicable Diseases (NCDs).^8^ Actions are planned to meet the goal of increasing the prevalence of physical activity in the population.^8^ However, sedentary behavior is disregarded as an important outcome in health conditions.^9^ Sedentary behavior and physical activity form patterns that may be reflected in health in different ways.^10^ Objective measurements of physical activity and sedentary time may be useful for ascertaining cutoff points within these behaviors as risk factors for occurrence of cardiometabolic multimorbidity. This has a significant impact on guidance for healthy behaviors at the population level, as a form of prevention and for health promotion.^11^^,^^12^

Physical activity recommendations (150 to 300 minutes per week at moderate intensity) for prevention of cardiovascular disease in the elderly and in people with chronic conditions are well established.^13^ Likewise, the amount of time spent on sedentary behavior has been shown to be a harmful factor proportional to the presence of the disease.^14^ It has been recommended that sedentary time should be replaced by physical activities of any intensity.^13^ Although the importance of achieving the physical activity recommendations is clear, some evidence of precisely how much time should be spent on PA and SB would guide proposals for attenuating the risk of having more than one chronic condition, seen in clinical practice.

Receiver operating characteristic (ROC) curve analysis is an important resource for ascertaining the presence or absence of cardiometabolic risks.^15^^,^^16^ In previous studies, some cutoff points for physical activity and sedentary behavior for disease prediction were proposed, and questionnaires were used to measure these behaviors.^17^ Subjective measurements may underestimate the accumulated time spent on sedentary behavior and overestimate the time spent on physical activity among older people, even though such measurements are easy to apply and have low cost. However, objective tools such as accelerometers provide reliable and valid physical activity measurements that make it possible to more faithfully capture different intensities of movement (sedentary, light, moderate and vigorous).^18^

## OBJECTIVE

Therefore, the aim of the present study was to identify the predictive power of different intensities of physical activity and sedentary behavior in relation to non-simultaneous occurrence of cardiovascular disease and diabetes in elderly men and women with one of the diagnosed diseases.

## METHODS

This was a population-based longitudinal study, carried out in the urban area of Florianópolis (SC), that used data from a study named EpiFloripa Idoso.^19^ That study was based on home interviews and had the aim of investigating “the relationships between cognitive and functional status, and violence and general conditions of health in the elderly aged 60 years and over”.^19^ EpiFloripa Idoso was approved by the Ethics Committee for Research on Human Beings (CEPSH) of the Federal University of Santa Catarina (Universidade Federal de Santa Catarina, UFSC) under registration number 352/2008, dated July 8, 2013.

The first wave of data (baseline) was collected between September 2009 and June 2010. The sample size was estimated based on a prevalence calculation according to the size of the population size over 60 years of age (4,460). From this calculation, the minimum number of elderly people to be interviewed was determined to be 1,599. For this, we used a 95% confidence level, sampling error of four percentage points, unknown prevalence of the phenomenon (50%) and a cluster design effect estimate of two. A further 20% were added for possible estimated losses and 15% for the purpose of testing associations. Through these design effects, while taking into account the availability of funding, the final sample was expanded to 1,911 elderly people.^19^

The sampling method used consisted of two-stage conglomerates, in which the first step was to select census tracts and the second, residential households. We identified 420 urban and residential census tracts, among which 80 were systematically drawn, considering the average monthly family income. Each census tract consisted of 61 to 725 households. Tracts with fewer than 150 households were grouped and those with more than 500 households were divided according to their location and income in order to reduce the coefficient of variation (52.7% for 80 sectors, to 35.2% for 83 sectors). For the second stage, it was considered that the average number of residents per household was 3.1 and that the individuals in the study age group corresponded to 11% of the population.^20^ Thus, it was estimated that it would be necessary to visit 60 households per sector from the list of addresses in order to find 20 seniors. All the elderly residents in the households selected were considered eligible, except for institutionalized elderly people who were housed in nursing homes, hospitals or prisons.^20^

The second wave sample (2013-2014) was composed of the elderly people (over 60 years) who had participated in the first wave. To form the second wave sample, home visits and calls were made and posters were put up, with the purpose of inviting the participants of the first wave of the study (1,705) to participate in this second stage. The elderly people who were not found after three visits on different days and at different times, or who refused to participate, had relocated, had become hospitalized or had died were excluded. Consequently, a final sample of 1,197 participants remained in the study.^19^ For the present study, data from this stage of EpiFloripa were used.

After potential participants had received explanations about the research and had agreed to participate through signing an informed consent statement, face-to-face interviews were conducted and a questionnaire was filled out. The participants were then invited to attend the Health Sciences Center facilities for the clinical examination and accelerometry stage. Six hundred and four elderly people participated in this stage and, among these, 484 agreed to use an accelerometer. Seventy-one individuals with reduced mobility or with any disability (intellectual, physical and/or sensory) that impeded them from using accelerometers were excluded. The remaining 49 people, who would have been eligible to use an accelerometer, were excluded due to a technical error.

Accelerometer data were collected using the GT3X or GT3X+ models (Actigraph, Pensacola, Florida, United States) and were analyzed using the Actilife software (Actigraph, Pensacola, Florida, United States). The participants were instructed to use the accelerometer for seven consecutive days and could remove it for sleep and in situations where the monitor would come into contact with water (e.g. bath, pool or beach). The device was attached to an elastic belt and fixed to the right hip above the iliac crest. On the second and fifth day of use of the accelerometer, telephone calls were made as a form of quality control. The accelerometers were programmed for a data sampling frequency of 30 Hz and these data were analyzed in 60-second windows. For data to be considered valid, the accelerometer needed to be used for four days a week (10 hours/day; or for weekend days, 8 hours/day). Consecutive zero periods of 60 minutes or more (with 2 minutes of tolerance) were considered to be non-use time and were excluded from the analysis.^21^ The cutoff points for the intensities of physical activity were taken from the model of Freedson et al.,^22^ as follows: light physical activity (LPA) (100-1951 counts/minute), moderate-to-vigorous physical activity (MVPA) (≥ 1952 counts/minute) and sedentary behavior (SB) (0-99 counts/minute). The minutes/week data of the variables were adjusted for the number of days of use. Valid data from accelerometer use were obtained from 425 elderly people (87.8%).

Sociodemographic variables (gender, age in years, marital status and education) came from a questionnaire. Information on the outcome of the present study, i.e. the multimorbidity variable for cardiometabolic diseases, was collected from the participants by means of a self-reported questionnaire, based on the following questions: “Has any doctor or healthcare professional ever said that you have or have had heart or cardiovascular disease?” and “Has any doctor or healthcare professional ever said that you have or have had hypertension (high blood pressure)?”; and for diabetes: “Has any doctor or healthcare professional ever said that you have or have had diabetes?” The answer options were yes or no, thus indicating the presence or absence of morbidities. People were considered to have cardiovascular diseases if they self-reported having a diagnosis of heart or cardiovascular disease and/or hypertension. The exposure variables, i.e. LPA, MVPA and SB, were defined in minutes/week and minutes/day through objective measurement using accelerometers.

Statistical analysis was performed using descriptive analysis with absolute and relative frequencies, 95% confidence intervals (95% CI), means and standard deviations, and medians and interquartile ranges (IQR). For the inferential analysis, receiver operating characteristic (ROC) curves were applied. ROC curves are a data attribute that is used to determine cutoff points in diagnostic or screening tests.^23^ The area under the ROC curve provides an assessment of the discriminatory power of PA intensities for determining absence of cardiometabolic multimorbidity and the power of SB for determining its occurrence. Multimorbidity was considered to be the occurrence of cardiovascular disease in the presence of diabetes, or the occurrence of diabetes in the presence of cardiovascular disease. The construction of the ROC curve was given by the positioning of the sensitivity on the y axis as a function of [1 - specificity] on the x axis.^23^ Sensitivity reports the percentage of affirmative outcomes that were correctly diagnosed using the indicator (true positives), while specificity describes the percentage of individuals who did not present the outcome and were correctly diagnosed using the indicator (true negatives). Thus, the values below the ROC curve represented the balance of the specificity and sensitivity pairs with all possible combinations. In determining a cutoff point, expressed as a bisector variable, the value of 0.5 was considered to be undetectable and 1.0, perfect detection.^24^ Thus, for the times spent on LPA, MVPA and SB to be considered to be significant predictors of cardiometabolic multimorbidity, the lower limit of the confidence interval was taken to be greater than or equal to 0.50.^24^ The 95% CI also determined predictive values. The analyses were stratified according to sex and whether or not diabetes and cardiovascular disease were present. The data were analyzed using the Stata software, version 13.0 (Stata Corporation, College Station, United States).

## RESULTS

The study included 425 elderly people, and 65.0% of them were women. The average age of the men was 73 years (standard deviation ± 7.41). Most of them reported having a partner (83.1%) and their mean schooling level was 9 years (standard deviation ± 6.7). The women’s mean age was 74 years (standard deviation ± 7.4). A majority of them were living without a partner (60.2%) and their mean schooling level was 7 years (standard deviation ± 5.2). The median LPA was 1,851 minutes/week (IQR: 1,358-2,283) among men and 1,887 minutes/week (IQR: 1435-2362) among women. The median MVPA was 121 minutes/week (IQR: 50-221) among men and 66 minutes/week (IQR: 18-133) among women. The median SB was 3,930 minutes/week (IQR: 3121-4746) among men and 3,688 minutes/week (IQR: 2971-4490) among women.

The values for the area under the ROC curve for the times spent on the PA and SB that had discriminatory power regarding cardiometabolic multimorbidity are presented in Table 1. The area under the ROC curve among men with diabetes that had predictive value was found within MVPA, with a value of 0.75 (95% CI: 0.538-0.962). Among women with diabetes, the area under the ROC curve with predictive value was found within MVPA with a value of 0.71 (95% CI: 0.524-0.886). Neither men nor women with cardiovascular disease presented areas under the ROC curve with predictive value or discriminatory power.

**Table 1.** ROC curve referring to weekly times spent doing different intensities of physical activity and sedentary behavior, for predict the presence or absence of cardiometabolic multimorbidity among elderly people, 2014 (n = 425)

Groups	Men (n = 160)				Women (n = 265)			
	Area under the ROC curve	95% CI of area	Sensitivity (%)	Specificity (%)	Area under the ROC curve	95% CI of area	Sensitivity (%)	Specificity (%)
With diabetes								
LPA	0.724	(0.326-1.000)	75.0	75.9	0.547	(0.310-0.784)	55.6	56.3
MVPA	0.750^*^	(0.538-0.962)	75.0	62.1	0.705^*^	(0.524-0.886)	66.7	65.6
SB	0.681	(0.284-1.000)	55.2	50.0	0.519	(0.273-0.766)	56.3	55.6
With cardiovascular diseases								
LPA	0.692	(0.277-1.000)	75.0	74.4	0.474	(0.228-0.721)	44.4	44.6
MVPA	0.587	(0.274-0.900)	50.0	51.2	0.454	(0.239-0.670)	44.4	42.9
SB	0.591	(0.482-0.699)	58.6	58.3	0.450	(0.187-0.714)	44.4	44.6

^*^Lower limit of confidence interval ≥ 0.50). LPA = light physical activity; MVPA = moderate-to-vigorous physical activity; SB = sedentary behavior; 95% CI = 95% confidence interval; ROC = receiver operating characteristic.

**Figure 1.**
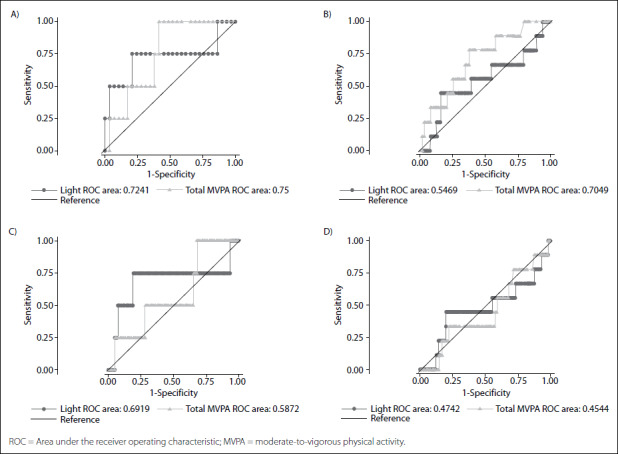
ROC curve describing weekly time spent engaging in different levels of physical activity as a preventive discriminator for cardiovascular disease occurrence in men (A) and women (B) with diabetes; and for diabetes in men (C) and women (D) with cardiovascular disease, 2014 (n = 425).

**Figure 2.**
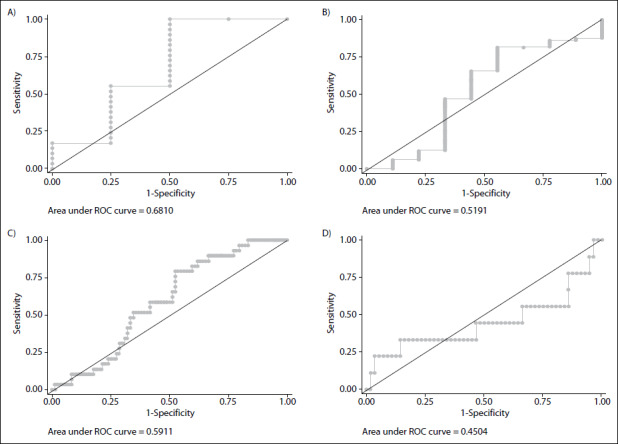
Area under the receiver operating characteristic (ROC) curve describing weekly time spent engaging in sedentary behavior as a risk discriminator for cardiovascular disease occurrence in men (A) and women (B) with diabetes; and for diabetes in men (C) and women (D) with cardiovascular disease, 2014 (n = 425).

Among men and women with diabetes, the time spent on MVPA discriminated with regard to presence or absence of cardiovascular disease (Figure 1). Among elderly people of both sexes with cardiovascular diseases, there was no predictive length of time for physical activity (Figure 1). Lastly, SB did not have predictive value for either men or women regarding the risk of cardiometabolic multimorbidity among those with diabetes and cardiovascular disease (Figure 2).

The values that were found to discriminate cutoff points for time spent on MVPA time, between presence and absence of multimorbidity, are highlighted in Table 2. Among the elderly participants with diabetes, it was seen that approximately 17 minutes of MVPA per day for men and 10 min per day for women were required for absence of multimorbidity.

**Table 2.** Time values and cutoff points for the estimated weekly and daily times spent engaging in moderate-to-vigorous physical activity, for predicting occurrence of cardiometabolic multimorbidity among elderly people in southern Brazil, 2014 (n = 425)

Groups	Men (n = 160)			Women (n = 265)		
	MD	Minutes^*^	Cutoff point^**^	MD	Minutes^*^	Cutoff point^**^
With diabetes						
MVPA	179	122	17.4	91	67	9.6

^*^Per week; ^**^Minutes per day. MVPA = moderate-to-vigorous physical activity; MD = median.

## DISCUSSION

The results from this study showed that only time spent on MVPA showed discriminatory power for presence or absence of cardiometabolic multimorbidity among elderly people with diabetes, of both sexes. The cutoffs for absence were minimums of approximately 17 minutes/day for men and 10 minutes/day for women. LPA and SB did not present predictive value for cardiometabolic multimorbidities in the elderly.

The daily MVPA cutoff point for absence of multimorbidity among elderly people with diabetes of both sexes was lower than the PA recommendations (150 to 300 minutes/week for moderate activities and 75 to 150 minutes/week for vigorous activities), based on subjective measurements for PA.^13^ The differences in these findings may be due to the type of measurement for PA evaluation. The data of the present study were based on accelerometer measurements, which are considered to be the gold standard for analyses.^9^

Thus, the present study suggests that women with diabetes who accumulate at least 9.6 minutes/day or 67 minutes/week of MVPA are at lower risk of cardiometabolic multimorbidity; and that men will achieve this with at least 17.4 minutes/day or 122 minutes/week. These findings are concordant with the recommendation that any duration of physical activity is reflected in healthy outcomes.^13^ For elderly people with diabetes, there is evidence showing that MVPA can reduce microvascular complications,^25^ such as retinopathy, nephropathy and neuropathies, which are inherent to the disease.^26^ Hence, the cutoff points identified in the present study may act as a facilitator for engaging elderly people in MVPA and, thus, minimizing the incidence of diseases affected by occurrence of multimorbidities.^27^

Our findings did not identify predictive values for time spent on LPA and SB for absence of multimorbidity for either sex, diagnosed either with diabetes or with cardiovascular disease. However, there is evidence showing the preventive effect of LPA on multimorbidities, as well as on improvement of functional capacities,^28^ and its significant clinical effects on blood pressure, body weight and glucose among the elderly.^29^

Regarding SB, it has been recognized in the literature that long times spent on SB pose risks of multimorbidity,^30^ regardless of PA engagement.^14^ Although there are no cutoff points for SB, recommendations for replacing it with any level of physical activity have been shown to result in health benefits.^13^ A study among elderly women showed that a transfer of 30 minutes from SB to MVPA reduced body mass index by 1.5 kg/m^2^.^31^ In the case of elderly people diagnosed with cardiometabolic disease, this effect may contribute to lifestyle improvements^9^^,^^27^ and mental health,^11^ as well as circumventing the effects of biological and genetic risks in this population.^27^ It is possible that in this group, LPA and SB were not predictive because of the diseases considered, which are more responsive to high-intensity actions, such as MVPA. It is known that among elderly people who reach light levels of physical activity in at least one weekly practice, the prevalence of multimorbidity is lower; however, this reduction is proportional to the increase in activity intensity.^32^

The strength of the present study is that makes a contribution to the small amount of evidence among elderly people in which objective measurement of PA were used.^9^ Its practical application is that the cutoffs presented can assist healthcare professionals, researchers and managers in planning and implementing interventions to reduce evidence-based disorders with multimorbidity. Moreover, these data contribute to both population-based and clinical contexts and may guide prescription of PA as a means for complementing treatment of these diseases.

However, some caveats are necessary in reading these data. They refer to a population-based sample residing in a region of Brazil with a high human development index (0.847), compared with the national average (0.727).^33^ The self-reporting of diseases may have been biased, given that this was dependent on recalling a doctor’s diagnosis. However, we excluded elderly people from our sample if they had a diagnosis of dementia or cognitive problems and would be unable to use an accelerometer. Lastly, multimorbidity was considered only in terms of cardiovascular disease (combined with hypertension) and diabetes, which limits comparability with other studies in which additional diseases were considered in the investigation.

## CONCLUSIONS

The accumulated time that elderly people with diabetes spent on MVPA was a predictor of cardiometabolic multimorbidity. Therefore, as a practical application in the future, it is recommended that elderly men with diabetes should perform moderate-to-vigorous physical activity for at least 17 minutes/day, and elderly women with diabetes for 9.6 minutes/day, to predict absence of multimorbidity. LPA and SB were not predictors for the absence of multimorbidity in either sex for diagnoses of either diabetes or cardiovascular diseases.
